# The Good School Toolkit for reducing physical violence from school staff to primary school students: a cluster-randomised controlled trial in Uganda

**DOI:** 10.1016/S2214-109X(15)00060-1

**Published:** 2015-07

**Authors:** Karen M Devries, Louise Knight, Jennifer C Child, Angel Mirembe, Janet Nakuti, Rebecca Jones, Joanna Sturgess, Elizabeth Allen, Nambusi Kyegombe, Jenny Parkes, Eddy Walakira, Diana Elbourne, Charlotte Watts, Dipak Naker

**Affiliations:** a London School of Hygiene and Tropical Medicine, London, UK; b Raising Voices, Kampala, Uganda; c Institute of Education, University College London, London, UK; d Makerere University, Kampala, Uganda

## Abstract

**Background:**

Violence against children from school staff is widespread in various settings, but few interventions address this. We tested whether the Good School Toolkit—a complex behavioural intervention designed by Ugandan not-for-profit organisation Raising Voices—could reduce physical violence from school staff to Ugandan primary school children.

**Methods:**

We randomly selected 42 primary schools (clusters) from 151 schools in Luwero District, Uganda, with more than 40 primary 5 students and no existing governance interventions. All schools agreed to be enrolled. All students in primary 5, 6, and 7 (approximate ages 11–14 years) and all staff members who spoke either English or Luganda and could provide informed consent were eligible for participation in cross-sectional baseline and endline surveys in June–July 2012 and 2014, respectively. We randomly assigned 21 schools to receive the Good School Toolkit and 21 to a waitlisted control group in September, 2012. The intervention was implemented from September, 2012, to April, 2014. Owing to the nature of the intervention, it was not possible to mask assignment. The primary outcome, assessed in 2014, was past week physical violence from school staff, measured by students' self-reports using the International Society for the Prevention of Child Abuse and Neglect Child Abuse Screening Tool—Child Institutional. Analyses were by intention to treat, and are adjusted for clustering within schools and for baseline school-level means of continuous outcomes. The trial is registered at clinicaltrials.gov, NCT01678846.

**Findings:**

No schools left the study. At 18-month follow-up, 3820 (92·4%) of 4138 randomly sampled students participated in a cross-sectional survey. Prevalence of past week physical violence was lower in the intervention schools (595/1921, 31·0%) than in the control schools (924/1899, 48·7%; odds ratio 0·40, 95% CI 0·26–0·64, p<0·0001). No adverse events related to the intervention were detected, but 434 children were referred to child protective services because of what they disclosed in the follow-up survey.

**Interpretation:**

The Good School Toolkit is an effective intervention to reduce violence against children from school staff in Ugandan primary schools.

**Funding:**

MRC, DfID, Wellcome Trust, Hewlett Foundation.

## Introduction

Exposure to physical violence in childhood is widespread and associated with increased risk of depressive disorders and suicide attempts,^1^ poor educational attainment,^2^ and increased risk of perpetrating or experiencing intimate partner violence in later relationships.^3^, ^4^ Recent national surveys suggest that, at least in some settings, violence from school staff could be an important but overlooked contributor to the overall health burden associated with violence against children. More than 50% of men and women reported physical violence from teachers when they were aged 0–18 years in Tanzania,^5^ and in Kenya more than 40% of 13–17-year-olds reported being punched, kicked, or whipped by a teacher in the past 12 months; 13–15% had experienced the same from a parent.^6^ There are no nationally representative data in Uganda, but our own work in one district shows that more than 90% of children aged about 11–14 years report lifetime physical violence from school staff, with 88% reporting caning, and 8% reporting extreme physical violence such as ever being choked, burned, stabbed, or severely beaten up.^7^ 4% had ever sought medical treatment for an injury inflicted by a staff member.^7^ In Uganda, corporal punishment has been banned by the Ministry of Education and Sports since 1997, although it is not fully illegal.

Assessments of interventions to reduce physical violence from school staff in low-income and middle-income settings are almost entirely absent from the literature.^8^ One study in Jamaica that tested the Incredible Years intervention in preschools showed a large reduction in negative teacher behaviours^9^ and improvements in child conduct disorder,^10^ suggesting that it is possible to change teachers' violent behaviour; we are not aware of any other trials on the topic.

**Research in context**

**Evidence before this study**

We are not aware of any other trials of interventions which seek to reduce physical violence from school staff towards primary school children. Existing interventions to prevent violence in schools come mainly from high-income countries and have largely focused on childhood sexual abuse, bullying, and other violence between students, with less emphasis on violence from school staff. A large global systematic review of school and school environment interventions on a range of health outcomes found no studies that addressed physical violence from school staff to students (searches to 2010). We did a systematic search of Medline, Embase, and ERIC from first record until January, 2013, and searched websites of various non-governmental organisations working on child protection (Unicef, Save the Children), and found no trials. We have done updated searches in Medline from Jan 1, 2013, to Jan 13, 2015, with MeSH terms and keyword searches using the terms “corporal punishment”, “physical violence”, “school”, and the clinical trial filter options, and have found no trials. Despite this lack of tested interventions, prevalence data indicate an unmet need. Where national surveys have been done in Kenya and Tanzania, they suggest that more than 50% of adolescents have experienced of physical violence from school staff.

**Added value of this study**

To our knowledge, this is the first trial of an intervention to reduce physical violence from school staff to primary school children. We therefore provide the first rigorous evidence that reducing this form of child maltreatment is possible.

**Implications of all the available evidence**

Our results suggest that the Good School Toolkit can reduce physical violence from school staff to primary school children in Uganda. Further research is needed to explore the effectiveness of this intervention over longer time periods, at scale, and to explore other types of interventions to reduce this common form of child maltreatment.

We report results of the Good Schools Study, which assessed the Good School Toolkit developed by the Ugandan not-for-profit organisation Raising Voices. Our main objective was to determine whether the Toolkit could reduce physical violence from school staff to students.

## Methods

### Setting

The Good Schools Study took place in 42 primary schools in Luwero District, Uganda, from January, 2012, to September, 2014. Luwero District is demographically similar to the rest of Uganda, according to the last Ugandan census in 2002. The intervention was implemented over 18 months, between September or October, 2012, and April or May, 2014. The study consisted of a cluster-randomised controlled trial, a qualitative study, an economic evaluation, and a process evaluation. The study was approved by the London School of Hygiene and Tropical Medicine Ethics Committee (6183) and the Uganda National Council for Science and Technology (SS2520). Our protocol is registered at clinicaltrials.gov (NCT01678846) and is published elsewhere,^11^ and we present our main trial results here.

### Design and participants

We did a two-arm cluster-randomised controlled trial with parallel assignment. A cluster design was chosen because the intervention operates at the school level. Using the official 2010 list of all 268 primary schools in Luwero as our sampling frame, we excluded 105 schools with fewer than 40 registered Primary (P) 5 students (aged around 10 years) and 20 schools with existing governance interventions implemented by Plan International. The remaining 151 schools were stratified into those with more than 60% girls, between 40 and 60% girls and boys, or more than 60% boys. 42 schools were randomly selected using a random number generator in Stata, proportional to size of the stratum. 42 was chosen on the basis of the number of schools in which Raising Voices could implement the intervention that would also give us power to detect a reasonably sized intervention effect. Stratified block randomisation was then used to allocate the schools to the two groups of the trial. Allowing for a loss to follow-up of two schools per group, and conservatively assuming interviews with 60 students per school, with a prevalence of past week physical violence of 50%^7^ and an intracluster correlation coefficient of 0·06 (from our baseline survey),^7^ we had 80% power to detect a 13% difference in the prevalence of reported violence between the intervention and control groups with 5% statistical significance. All headteachers agreed for their schools to participate in the study and schools were enrolled by Raising Voices staff and JC.

Cross-sectional baseline and endline surveys were conducted at schools in June or July, 2012, and June or July, 2014, respectively. We chose this design rather than a cohort design to avoid problems related to attrition of individual students, and because our main aim was to measure prevalence at follow-up. Parents were notified and could opt children out, but children themselves provided consent. Up-to-date lists of all P5, 6, and 7 students (aged about 11–14 years) were obtained from each school, and a simple random sample of up to 130 P5, 6, and 7 students (selected using a random number generator in Stata) were invited for individual interviews where surveys were administered. If there were fewer than 130 P5–7 students in a school, all were invited for interview. Implementation of the intervention was school-wide, but data was collected from P5–7 students only, because they were able to respond to questions in survey format. All those who could speak Luganda or English and who were deemed by interviewers to be able to understand the consent procedures were eligible. All school staff were invited to participate and provided informed consent. At least one repeat visit was made to find staff and sampled students absent on the day of the survey.

### Procedures

Schools were stratified into 12 blocks on the basis of whether they were urban or rural, whether their baseline prevalence of past week violence was above or below the baseline median of past week physical violence from school staff of 55%, and a qualitative assessment of high or low likelihood of attrition from the trial. LA used block randomisation to generate allocation lists. At a meeting of all school headteachers, a representative from each school within a block was invited to place their school name in an opaque bag. Names were then drawn from the bag by a headteacher nominated by the group and schools were then allocated to intervention (the Good School Toolkit) or wait-list control conditions (usual practice) in the sequence on the allocation list, recorded by JC. Owing to the nature of the intervention, it was not possible to mask participants. Allocation was not intentionally revealed to those collecting data; however, given the nature of the intervention, they should also be regarded unmasked. The statistician (RJ) was masked to allocation while performing analyses.

Potential risks related to the intervention itself were minimal, but we anticipated that during survey data collection we would detect children in need of support from child protective services. Children were informed during the consent process that their details might be passed on to child protection officers. Referrals were based on predefined criteria agreed with service providers, related to the severity and timing of violence reported.^12^ All children were offered counselling regardless of what they disclosed. Any adverse effects of the intervention itself were monitored during regular visits to schools by the dedicated study monitoring officer (AM). Monitoring data were collected termly via structured classroom observations, formal and informal interviews with staff, and observations of students.

No major changes to the trial protocol were made. We made changes to our child protection referral strategy after the baseline survey. These experiences at baseline are published elsewhere,^12^ and will be described in a separate publication for the follow-up.

The Good School Toolkit is a complex behavioural intervention which aims to foster change of operational culture at the school level. The Toolkit draws on the Transtheoretical Model,^13^ and contains behavioural change techniques that have been shown to be effective in a variety of fields^14^ and have been included in interventions to change teacher behaviour in primary schools^9^, ^15^ and to reduce perpetration of intimate partner violence.^16^ The Toolkit materials consist of T-shirts, books, booklets, posters, and facilitation guides for about 60 different activities. These activities are related to creating a better learning environment, respecting each other, understanding power relationships, using non-violent discipline, and to improving teaching techniques. More details are provided in the panel.

Panel **The Good School Toolkit intervention by Raising Voices**

**Materials and content**

Specific behaviour-change techniques for staff, students, and administration include: setting school-wide goals, developing action plans with specific dates for deliverables, encouraging empathy by facilitating reflection on experiences of violence, providing new knowledge on alternative non-violent discipline, and providing opportunities to practise new behavioural skills. Schools are encouraged to self-monitor their progress according to their action plans. Reinforcement of new information and ideas, feedback on progress, and modelling of new techniques and behaviours is provided by visits from the Raising Voices team, and also within school by “protagonists” to their peers as they gain new knowledge and skills. Children participate actively and form committees and groups related to different activities. Schools reward successful achievement of their goals and action plan deliverables by creating celebrations. Social support for behavioural change is also created because the intervention engages multiple groups within a school (teachers, administration, students, and also parents) to change ideas and attitudes.

**Procedures**

After a school's agreement to participate, an inception visit of 2 hours is held where Raising Voices introduces the Toolkit to all school staff. Once the schools are committed to implementing the Toolkit, at least two staff protagonists are identified who attended a 3-day residential workshop. During this workshop, the protagonists become familiar with the Toolkit, and develop an action plan for their school. Raising voices staff members then provide direct one-on-one support to key staff protagonists and at least two key student protagonists in each school to carry out the action plan. The Toolkit itself has six steps, which are designed to be implemented in sequence to bring schools through a process of change. Protagonists can choose which activities they implement, but should do a minimum number from each step before moving on to the next.

**Who provided**

Raising Voices staff members are trained facilitators and advocates, and have received approximately 100 h of training with individualised coaching support to understand the ideas and content of the Toolkit. The key protagonists in each school are not required to have any specific background or training, but receive the 3-day residential workshop and ongoing support.

**How**

During the intervention, Raising Voices staff members provide direct one-on-one support in the form of in-person visits and telephone calls to staff protagonists, and in-person visits to student protagonists. Staff and student protagonists conduct face-to-face activities with other staff and students in their school, mainly in groups. Children and staff members encourage others to form, lead, and join groups to do various intervention activities.

**Where**

Activities with students and staff were conducted in schools. Some activities involve creating a better school environment by painting murals on school walls, and hanging codes of conduct in visible places; however, the intervention does not require any physical infrastructure.

**When and how much**

Raising Voices staff made in-person visits to protagonists in each school on a quarterly basis, and telephoned school staff members approximately monthly, although this varied slightly depending on need. The Toolkit itself is designed to be implemented in a flexible fashion, and there are no prescribed number of activities or set schedule upon which they should be implemented. However, schools should proceed in sequence and conduct a minimum number of activities, which depends on the stage, prior to progressing to the next stage.

**Tailoring, modifications, fidelity**

The Toolkit is specifically designed to be flexible in implementation. Data on activities conducted by protagonists within schools was collected and cleaning and analysis of this is ongoing. Raising Voices staff visited each school at least once per school term, conducted a 3-day residential workshop with all teacher protagonists in January, 2013, met with protagonists to review progress after one to two terms of implementation in August, 2013, and held a meeting with protagonists so they could learn from each other in December, 2013.

### Outcomes

The primary outcome was past week physical violence from a school staff member, self-reported by students according to the International Society for the Prevention of Child Abuse and Neglect Screening Tool—Child Institutional (ICAST-CI).^17^ Secondary outcomes were safety and wellbeing in school, mental health status (Strengths and Difficulties Questionnaire),^18^ and scores on educational tests. All were measured with instruments widely used internationally which have been validated in a variety of settings (see appendix for details). Instruments were translated where necessary, and some items and time frames for recall added to the ICAST to capture the Ugandan context. All were pretested for understanding and piloted before the baseline survey. Analysis of our baseline data shows construct validity for our primary outcome in our sample, with children reporting past week physical violence also reporting high levels of mental health difficulties.

### Statistical analysis

We did an intention-to-treat analysis using data from our cross-sectional follow-up survey. All analyses were done in Stata/IC 13.1. Data were collected using a survey programmed into tablet computers with algorithms designed to eliminate erroneous skips. Most educational test data were collected on paper; all were double scored, and double entered. All trial outcomes were measured at the level of individual student participants. Analysis was done with individual level student data, accounting for clustering of students within schools using mixed-effects regression models. “Unadjusted” analyses of continuous outcomes control for the school-level mean of the outcome at baseline. Adjusted analyses control additionally for students' sex, whether or not they had a disability, and their school's location (urban or rural) and baseline level of past week physical violence from school staff (modelled as a continuous variable at the school level). For non-normally distributed continuous outcomes, 95% confidence intervals (CI) were estimated by use of 2000 bootstrap replications. All covariates were specified a priori. We planned a priori to conduct subgroup analyses by sex, by urban or rural location of the school, and by baseline levels of past week physical violence from school staff.

### Role of the funding source

None of the funding sources played a role in the design of the study, data collection, analysis, interpretation, or writing of the results. The corresponding author had full access to all the data in the study and had final responsibility for the decision to submit for publication.

## Results

42 schools participated in the baseline and endline survey, and 3814 (92·3%) of sampled students were interviewed at endline (figure). School, student, and staff characteristics were evenly distributed across study groups at baseline (table 1). Most students were aged 11–14 years (mean age 13·0 years [SD 1·5]), 52% were female, and 7·3% reported some form of disability. More than half reported eating fewer than three meals in the day before the survey. Staff were in their mid-30s, and nearly 60% were female. Demographic characteristics of staff and students at the endline survey are reported in the appendix.

**Figure:**
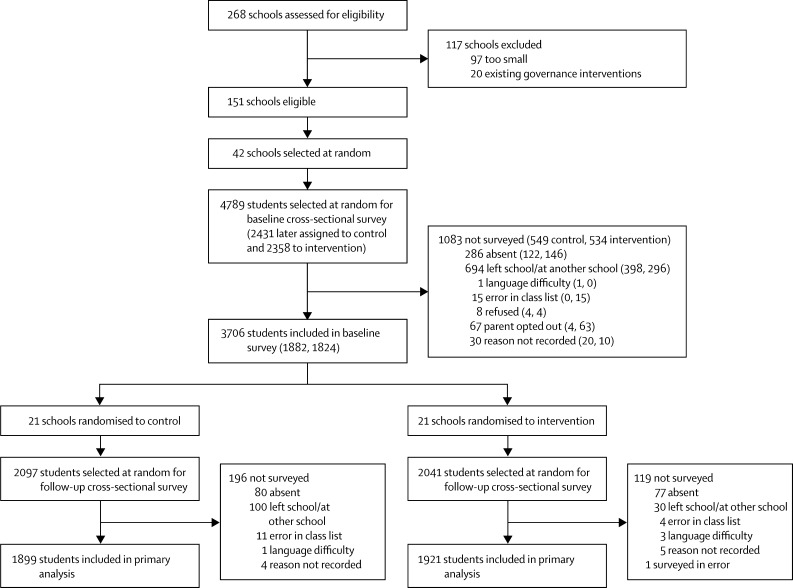
Trial profile

**Table 1:** Characteristics of schools, students, and staff at baseline

		**Control**	**Intervention**	**All schools**
**School characteristics**				
N		21	21	42
Urban location		8 (38·1%)	7 (33·3%)	15 (35·7%)
Prevalence of violence (%), mean (SD)*		54·3 (11·7)	52·8 (13·1)	53·6 (12·3)
**Student characteristics**				
N		1882	1824	3706
Age (years), mean (SD)		13·0 (1·5)	13·1 (1·5)	13·0 (1·5)
Sex				
	Female	1010 (53·7%)	927 (50·8%)	1937 (52·3%)
School class				
	5	703 (37·4%)	739 (40·5%)	1442 (38·9%)
	6	697 (37·0%)	644 (35·3%)	1341 (36·2%)
	7	482 (25·6%)	441 (24·2%)	923 (24·9%)
Disability				
	Some disability	142 (7·5%)	129 (7·1%)	271 (7·3%)
Meals eaten previous day				
	1 meal	250 (13·3%)	266 (14·6%)	516 (13·9%)
	2 meals	743 (39·5%)	700 (38·4%)	1443 (38·9%)
	3+ meals	888 (47·2%)	858 (47·0%)	1746 (47·1%)
Hours of work each day				
	Less than 1 h	788 (42·1%)	635 (34·9%)	1423 (38·6%)
	1–2 h	780 (41·7%)	868 (47·8%)	1648 (44·7%)
	More than 2 h	304 (16·2%)	314 (17·3%)	618 (16·8%)
Mode of transport to school				
	Other	62 (3·4%)	77 (4·3%)	139 (3·8%)
	Walking alone	440 (23·9%)	450 (25·2%)	890 (24·6%)
	Walking with someone you know	1161 (63·2%)	1153 (64·6%)	2314 (63·9%)
	Board at school	175 (9·5%)	104 (5·8%)	279 (7·7%)
Absence from school in previous week				
	1 or more days missed	347 (19·0%)	425 (23·5%)	772 (21·2%)
**Staff characteristics**				
N		304	273	577
Age (years), mean (SD)		35·1 (8·7)	33·8 (8·3)	34·5 (8·6)
Sex				
	Female	177 (58·2%)	161 (59·0%)	338 (58·6%)
Tribe				
	Muganda	196 (64·5%)	166 (60·8%)	362 (62·7%)
Religion				
	Roman Catholic	87 (28·7%)	74 (27·3%)	161 (28·0%)
	Anglican	94 (31·0%)	103 (38·0%)	197 (34·3%)
	Pentecostal	54 (17·8%)	42 (15·5%)	96 (16·7%)
	Seventh Day Adventist	34 (11·2%)	15 (5·5%)	49 (8·5%)
	Muslim	34 (11·2%)	37 (13·7%)	71 (12·4%)
Marital status				
	Single	64 (21·1%)	73 (26·9%)	137 (23·8%)
	In a relationship	23 (7·6%)	26 (9·6%)	49 (8·5%)
	Married / living together	204 (67·1%)	159 (58·7%)	363 (63·1%)
	Divorced / separated / widowed	13 (4·3%)	13 (4·8%)	26 (4·5%)
Housing				
	Own	112 (36·8%)	87 (32·0%)	199 (34·5%)
	Rented	91 (29·9%)	73 (26·8%)	164 (28·5%)
	Live somewhere without paying	25 (8·2%)	15 (5·5%)	40 (6·9%)
	Employer pays	74 (24·3%)	95 (34·9%)	169 (29·3%)
	Other	2 (0·7%)	2 (0·7%)	4 (0·7%)

All statistics are n (%) unless otherwise specified. There was slightly more missing data on school absence in the control than in intervention arm of the study (2·9% vs 0·9%). For all other measures, the quantity of missing data was low (<3% for students; <1% for staff) and similar in both study groups.

* Mean of school means of proportion of students reporting physical violence from school staff in the past week.

At baseline, levels of each primary and secondary outcome were similar across groups (table 2). 54% of students reported past week physical violence from school staff. The mean score on the Strengths and Difficulties Questionnaire (on a scale of 0 to 2) was 0·47 (SD 0·27), and the mean wellbeing score (on a scale of 0 to 15) was 10·9 (2·5). Educational test scores at baseline were comparable across groups, and showed low levels of reading ability.

**Table 2:** Primary and secondary outcomes at baseline

	**Control (1882 students; 304 staff)**	**Intervention (1824 students; 273 staff)**	**All schools (3706 students; 577 staff)**
**Physical violence**			
Student self-reported past week physical violence at school	54·6% (1028/1882)	52·7% (962/1824)	53·7% (1990/3706)
School staff self-reported past week use of physical violence	43·1% (131/304)	39·9% (109/273)	41·6% (240/577)
**Mental health and wellbeing**			
SDQ total difficulties score	0·47 (0·26)	0·46 (0·27)	0·47 (0·27)
School wellbeing	10·9 (2·5)	10·9 (2·5)	10·9 (2·5)
**Educational performance**			
Word recognition in English (words per min)	47·0 (24·8)	45·3 (25·8)	46·2 (25·3)
Word reading in English	49·8 (18·3)	47·2 (20·5)	48·5 (19·4)
Reading comprehension in English	3·1 (1·5)	2·9 (1·5)	3·0 (1·5)
Word recognition in Luganda (words per min)	23·8 (15·7)	22·6 (15·9)	23·2 (15·8)
Word reading in Luganda	27·1 (18·5)	25·9 (18·8)	26·5 (18·7)
Reading comprehension in Luganda	2·4 (1·5)	2·1 (1·5)	2·2 (1·5)
Silly sentences test	20·1 (10·1)	21·2 (11·7)	20·6 (10·9)
Spelling in English	7·9 (5·0)	8·0 (6·0)	8·0 (5·5)
Written numeracy	24·4 (7·3)	25·0 (7·7)	24·7 (7·5)

Data are % (n/N) and mean (SD). There were slightly more missing data on reading comprehension in Luganda in the control than in the Intervention arm (12·2% *vs* 9·3%). More than 8% of students had missing data for word reading in Luganda, but the quantity of missing data was similar in both study arms (8·7% *vs* 8·4%). For all other measures, the quantity of missing data was low (<6%) and similar in both study arms. For the educational performance assessments which were administered at class level (silly sentences, spelling in English, and written numeracy) data from a total of 5786 students was included in the analysis (control 3139; intervention 2647). Students' experience of past term physical violence is not reported, as this question was not asked at baseline.

At follow-up, 80·7% of students in the intervention group had completed the previous grade in the same school that they were currently in, and 89·1% of staff had worked in their current school for more than 1 year and thus would have been exposed to at least some intervention activities. Levels of absenteeism were high, with about 20% of students surveyed indicating that they had been absent in the past week. During the trial, we recorded one major incident of contamination, where an intervention school invited head teachers from three neighbouring control schools to an event about the Toolkit. The control schools did not do any further activities and did not receive any support from Raising Voices, and the intervention school was asked not to invite other schools to its events until after the trial.

In the follow-up cross-sectional survey, 595 of 1921 students (31·0%) in the intervention group reported past week physical violence from school staff, versus 924 of 1899 students (48·7%) in the control group (table 3). After accounting for clustering between students within schools, there was a 60% reduction in the odds of our physical violence outcome. This corresponds to a 42% reduction in risk of past week physical violence from school staff.

**Table 3:** Effect of the intervention on outcomes

	**Summary statistics**		Intervention effect*	
	Control (1899 students; 308 staff)	Intervention (1921 students; 283 staff)	Basic model	Adjusted model
**Physical violence**				
Student self-reported past week physical violence at school	48·7% (924/1899)	31·0% (595/1921)	0·40 (0·26 to 0·64) p<0·0001	0·39 (0·25 to 0·62) p<0·0001
Student self-reported past term physical violence at school	80·5% (1528/1899)	60·2% (1157/1921)	0·32 (0·18 to 0·55) p<0·0001	0·31 (0·18 to 0·53) p<0·0001
School staff self-reported past week use of physical violence	32·5% (100/308)	15·5% (44/283)	0·39 (0·20 to 0·73) p=0·0036	0·37 (0·20 to 0·69) p=0·0018
**Mental health and wellbeing**				
SDQ total difficulties score	0·44 (0·26)	0·44 (0·26)	0·01 (-0·02 to 0·04) 0·6585	0·00 (-0·03 to 0·03) 0·8907
School wellbeing	11·1 (2·5)	11·7 (2·4)	0·58 (0·25 to 0·91) 0·0006	0·59 (0·24 to 0·93) 0·0008
**Educational performance**				
Word recognition in English (words per minute)	49·7 (27·7)	48·6 (27·7)	0·16 (-3·47 to 3·79) 0·9308	0·27 (-3·48 to 4·02) 0·8873
Word reading in English	49·0 (21·3)	47·8 (21·3)	1·79 (-1·33 to 4·90) 0·2611	1·90 (-1·23 to 5·02) 0·2344
Reading comprehension in English	1·4 (1·5)	1·4 (1·6)	0·13 (-0·18 to 0·44) 0·4115	0·12 (-0·20 to 0·44) 0·4593
Word recognition in Luganda (words per minute)	33·2 (20·7)	31·2 (20·7)	-1·08 (-3·36 to 1·19) 0·3511	-0·96 (-3·40 to 1·48) 0·4413
Word reading in Luganda	37·9 (22·0)	35·1 (22·4)	-2·01 (-4·66 to 0·65) 0·1385	-1·89 (-4·67 to 0·90) 0·1844
Reading comprehension in Luganda	2·8 (1·8)	2·6 (1·8)	-0·10 (-0·31 to 0·11) 0·3569	-0·10 (-0·32 to 0·13) 0·3937
Silly sentences test	9·3 (5·2)	9·1 (5·1)	-0·63 (-1·61 to 0·35) 0·2106	-0·55 (-1·58 to 0·48) 0·2963
Spelling in English	10·8 (6·2)	10·6 (6·9)	-0·19 (-1·13 to 0·74) 0·6875	-0·17 (-1·15 to 0·80) 0·7260
Writen numeracy	24·9 (7·4)	24·7 (8·1)	-0·87 (-1·87 to 0·14) 0·0904	-0·91 (-1·99 to 0·17) 0·0972

* Odds ratio (95% CI), p value for physical violence; difference (95% CI), p value for all other outcomes. The quantity of missing data was extremely low for all measures (<0·5%) and similar in both study arms. The basic model for continuous outcomes (ie, SDQ, school wellbeing, and all educational outcomes) adjusted for baseline by including as a covariate in statistical models the school level mean of the outcome at baseline. The basic model for binary outcomes (ie, the three physical violence measures) made no adjustment for baseline. Adjusted models controlled for school location (uban *vs* rural) and school-level prevalence of physical violence at baseline. Adjusted models for student-level outcomes controlled additionally for students' sex and disability. The adjusted model for staff self-reported past week use of physical violence controlled additionally for sex. For the educational performance assessments which were administered at class level (silly sentences, spelling in English and written numeracy) data from a total of 5291 students were included in the analysis (control 2833; intervention 2458). Adjusted models for these three outcomes did not control for students' sex and disability, as these measures were not collected for all students in this wider sample. For non-normally distributed continuous outcomes 95% CI were estimated using the bootstrap method. All models accounted for correlations between students within schools. At follow-up, the ICC was 0·105 in the control arm.

The Strengths and Difficulties Questionnaire total scores did not differ between groups at follow-up, and were similar to baseline scores, indicating that there was no detectable effect of the intervention on this outcome after 18 months of implementation. Levels of school wellbeing were higher in the intervention than control group, however. There was no evidence that the intervention had an impact on any educational test scores (table 3).

There was weak evidence that the intervention had a stronger effect in male students (odds ratio [OR] 0·34, 95% CI 0·21–0·56) than female students (OR 0·46, 95% CI 0·29–0·74; p for interaction=0·043), but there was no evidence that the intervention effect differed by urban or rural location or baseline level of past week violence from school staff (see appendix).

We did supplementary analyses to examine students' self-reports of physical violence from school staff in the past term, and staff reports of their use of physical violence against students (table 3). Students in intervention schools reported lower levels of past term violence. Staff in the intervention group also reported using less violence in the past week than those in the control group.

No adverse effects of the intervention itself were detected via monitoring visits. At follow-up, 434 of 3820 children were referred because they disclosed severe violence in the survey (239 [12·6%] in the control group, 195 [10·2%] in the intervention group).

## Discussion

The Good School Toolkit produces a large reduction in physical violence from school staff, as reported by primary school students in Luwero District, Uganda. There was some evidence that the Toolkit was more effective for male than female students, although it was highly effective for both sexes. The Toolkit also improved students' feelings of wellbeing and safety at school, suggesting that the intervention is effective in changing the school environment. The Toolkit did not affect student mental health or student educational test scores.

Qualitative research shows the existence of norms supportive of “beating” as being necessary for positive child development in Tanzania^19^ and South Africa,^20^ as well as in some high-income settings such as the USA, where corporal punishment in schools is legal in 18 states.^21^ Change in attitudes and behaviours related to physical violence and punishment has occurred over several decades in Sweden, where 53% of parents supported corporal punishment of their children in 1965 versus 11% in 1994.^22^ Our results are highly encouraging because they demonstrate that it is possible to change an entrenched, normative behaviour such as the use of physical violence over the 18-month timescale of programme implementation.

The Toolkit also positively affected students' feelings of safety and wellbeing at school, but contrary to our hypotheses, we did not find effects on mental health outcomes or educational test scores. According to our theory of change, improvements in school wellbeing and reductions in mental health symptoms should precede improvements in educational outcomes.^11^ It is possible that, over the timescale of the intervention implementation, these effects simply did not take place. It is also possible that staff are more likely to punish students with worse symptoms of externalising or internalising disorders. If this is the case, we would not expect reducing violent punishment to automatically reduce mental health symptoms.

We also note that both mental health symptoms and educational outcomes are likely to be associated with a range of socioeconomic, familial, and structural factors outside of school, which might not be amenable to a school-based violence prevention programme. Most of the schools involved in the project, similar to other schools in Uganda, are faced with large structural issues related to poverty—for example, large class sizes, poor physical infrastructure, and a lack of resources for teaching. Although students felt safer in intervention schools, it might be that improving the atmosphere at school is necessary but not sufficient to improve outcomes in the short term of our evaluation.

Our study has a number of strengths, and also some limitations. Our results should be generalisable to other African settings—we sampled schools to be representative of larger schools in Luwero District, 100% of schools agreed to participate, and no schools dropped out of the study. However, students in Ugandan primary schools are slightly older than in some higher-income countries, which might affect the generalisability of our results to other primary school populations with different age profiles. We were well-powered to detect an effect, and had very high response rates and very low levels of missing data. We noted an instance of possible contamination during the trial, which would have biased effect estimates downwards, yet we still detected a large effect.

Although we have made use of a standardised, internationally recognised and widely used questionnaire to measure self-reported violence exposure, our main limitation is that violence measures are by necessity reported rather than observed. We used student reports of violence outcomes as the most conservative test of the intervention effect. Reports from school staff about use of physical violence against students are likely to be biased in the same direction as the intervention effect, whereas student reports would likely be biased in the opposite direction. Third-party corroborated reports of violence exposure vastly underestimate prevalence compared with self-report^23^ and medical record checks for injuries resulting from staff violence are not feasible in this context. Nonetheless, staff reports and students' reports of past-term exposure (when the research team was not in their school) show very similar effect sizes and direction, lending support to our results. Similar to other complex social interventions, we were unable to mask participants or data collectors to allocation. This could have introduced bias towards a larger effect, but it is unlikely that this would entirely account for such a large observed difference.

Given the prevalence of violence observed in our sample and in other surveys in the region, use of the Toolkit or similar programmes could have a major effect on the burden of child maltreatment in countries where violence from school staff is common. Further analyses are underway to explore the effect of the intervention on other forms of violence, including violence from peers.

We note that, although we observed a large reduction in levels of violence, absolute prevalence of physical violence still remained high at 30% and 60% in the past week and past term, respectively. Further research is needed to examine whether the Toolkit can further reduce prevalence if implemented over longer time periods, to examine whether the effects of the Toolkit are sustainable without ongoing support from Raising Voices, and to examine the intervention effect at scale. Further research to address violence happening outside of schools is also needed.
