# Supplementary material for: The Good School Toolkit for reducing physical violence from school staff to primary school students: a cluster-randomised controlled trial in Uganda
Source: Lancet Glob Health. 2015 Jul;3(7):e378–86. doi: 10.1016/S2214-109X(15)00060-1 (PMC4928210; doi:10.1016/S2214-109X(15)00060-1)
Supplement: Supplementary appendix [file mmc1.pdf]

THE LANCET Global Health

## Supplementary appendix

This appendix formed part of the original submission and has been peer reviewed. We post it as supplied by the authors.

Supplement to: Devries KM, Knight L, Child JC, et al. The Good School Toolkit for reducing physical violence from school staff to primary school students: a cluster-randomised controlled trial in Uganda. *Lancet Glob Health* 2015; **3:** e378–86.

# Appendix
# Good Schools Study

## Instruments used to measure outcomes

| Variable name | Instrument, Items | Coding |
|---|---|---|
| Physical violence, self-reported by students<br><br>Time frame: past week, past school term | International Society for the Prevention of Child Abuse and Neglect Screening Tool-Child Institutional[17] (ICAST-CI), modified to include time frames.<br><br>Has a school staff member: hurt you or caused pain to you? Slapped you with a hand on your face or head as punishment? Slapped you with a hand on your arm or hand? Twisted your ear as punishment? Twisted your arm as punishment? Pulled your hair as punishment? Hit you by throwing an object at you? Hit you with a closed fist? Hit you with a stick? Caned you? Kicked you? Knocked you on the head as punishment? Made you dig, slash a field, or do other labour as punishment? Hit your fingers or hands with an object as punishment? Crushed your fingers or hands as punishment? Made you stand /kneel in a way that hurts to punish you? Made you stay outside for example in the heat or rain to punish you? Burnt you as punishment? Taken your food away from you as punishment? Forced you to do something that was dangerous? Choked you? Tied you up with a rope or belt at school? Tried to cut you purposefully with a sharp object? Severely beat you up? | Coded 1 if answered yes to any of the items; 0 if answered no to all items. |
| Physical violence (use of), self-reported by staff members<br><br>Time frame: past week | Adapted from the International Society for the Prevention of Child Abuse and Neglect Screening Tool-Child Institutional[17] (ICAST-CI), modified to include time frames.<br><br>What are the methods of physical discipline you have used with students? Have you ever: Slapped them with a hand on their face or head as punishment? Twisted their ear as punishment? Twisted their arm as punishment? Pulled their hair as punishment? Hit them by throwing an object at them? Hit them with a closed fist? Hit them with a stick? Caned them? Kicked them? Knocked them on the head as punishment? Made them dig, slash a field, or do other labour as punishment? Hit their fingers or hands with an object as punishment? Crushed their fingers or hands as punishment? Made them stand/kneel in a way that hurts to punish them? Made them stay outside for example in the heat or rain to punish them? Burnt them as punishment? Taken their food away as punishment? Forced them to do something that was dangerous? Choked them? Tied them up (with a rope or belt) at school? Tried to cut them purposefully with a sharp object? Made them roll over on the ground until they were dizzy as punishment? | Coded 1 if answered yes to any of the items; 0 if answered no to all items. |
| Mental health symptoms | Strengths and Difficulties Questionnaire (SDQ)[18] (25 items) | Total difficulties score, divided by the number of completed items. Modelled as a continuous variable. Range 0 (no difficulties) to 2 (high difficulties). |
| Safety and well-being at school | Each question asked with response options all the time, most of the time, sometimes, never:<br>I feel that my teachers care about me. I feel safe in school. I feel like I belong at school. I like to spend time at school. I am scared of my teachers (reverse coded). | Scores summed, modelled as a continuous variable. Range 0 (low) to 15 (high). |
| Word recognition in | Early Grade Reading Assessment, Uganda version | Continuous variable. |

| | | |
|---|---|---|
| English(words per minute) | Number of words read correctly (out of a maximum of 50), divided by the time (out of a maximum of 60 seconds) | |
| Word reading in English | Early Grade Reading Assessment, Uganda version<br>Number of words read correctly out of 60, in 60 seconds. | Continuous variable. Range 0-68 |
| Reading comprehension in English | Early Grade Reading Assessment, Uganda version<br>Number of questions answered correctly out of 5 | Continuous variable. Range 0-5 |
| Word recognition in Luganda (words per minute) | Early Grade Reading Assessment, Uganda version<br>Number of words read correctly (out of a maximum of 50), divided by the time (out of a maximum of 60 seconds) | Continuous variable. |
| Word reading in Luganda | Early Grade Reading Assessment, Uganda version<br>Number of words read correctly out of 60, in 60 seconds. | Continuous variable. Range 0-61 |
| Reading comprehension in Luganda | Early Grade Reading Assessment, Uganda version<br>Number of questions answered correctly out of 5 | Continuous variable. Range 0-5 |
| Silly sentences test | Adapted from an intervention trial in Kenya<br>Number of correct responses out of 40 | Continuous variable. Range 0-40 |
| Spelling in English | Adapted from an intervention trial in Kenya<br>Number of correct responses out of 25 | Continuous variable. Range 0-25 |
| Written numeracy | Adapted from an intervention trial in Kenya<br>Number of correct responses out of 38 | Continuous variable. Range 0-38 |

## Supplementary table 1: Characteristics of students and staff in each study arm at follow up

| | Control | Intervention | All Students |
|---|---|---|---|
| **Student characteristics** | **N = 1,899** | **N = 1,921** | **N = 3,820** |
| Age (years) – mean (sd) | 12·9 (1·6) | 13·1 (1·5) | 13·0 (1·5) |
| **Sex** | | | |
| Female | 1018 (53·6%) | 1036 (53·9%) | 2054 (53·8%) |
| **School class** | | | |
| 5 | 678 (35·7%) | 748 (38·9%) | 1426 (37·3%) |
| 6 | 655 (34·5%) | 657 (34·2%) | 1312 (34·3%) |
| 7 | 566 (29·8%) | 516 (26·9%) | 1082 (28·3%) |
| **Disability** | | | |
| Some disability | 349 (18·4%) | 457 (23·8%) | 806 (21·1%) |
| **Meals eaten previous day** | | | |
| 1 meal | 262 (13·8%) | 337 (17·6%) | 599 (15·7%) |
| 2 meals | 729 (38·4%) | 780 (40·6%) | 1509 (39·5%) |
| 3+ meals | 905 (47·7%) | 803 (41·8%) | 1708 (44·8%) |
| **Hours of work each day** | | | |
| Less than 1 hour | 626 (33·0%) | 665 (34·9%) | 1291 (34·0%) |
| 1-2 hours | 938 (49·5%) | 910 (47·7%) | 1848 (48·6%) |
| More than 2 hours | 331 (17·5%) | 332 (17·4%) | 663 (17·4%) |
| **Mode of transport to school** | | | |
| Other | 75 (4·0%) | 100 (5·2%) | 175 (4·6%) |
| Walking alone | 446 (23·7%) | 433 (22·7%) | 879 (23·2%) |
| Walking with someone you know | 1133 (60·2%) | 1249 (65·4%) | 2382 (62·8%) |
| Board at school | 228 (12·1%) | 128 (6·7%) | 356 (9·4%) |
| **Absence from school in previous week** | | | |
| 1 or more days missed | 365 (19·3%) | 384 (20·1%) | 749 (19·7%) |
| **Staff characteristics** | **N = 308** | **N = 283** | **N = 591** |
| Age (years) – mean (sd) | 35·2 (9·1) | 34·7 (8·4) | 35·0 (8·7) |
| **Sex** | | | |
| Female | 200 (64·9%) | 168 (59·4%) | 368 (62·3%) |
| **Tribe** | | | |
| Muganda | 194 (63·0%) | 182 (64·3%) | 376 (63·6%) |
| Other | 114 (37·0%) | 101 (35·7%) | 215 (36·4%) |
| **Religion** | | | |
| Roman Catholic | 84 (27·5%) | 82 (29·0%) | 166 (28·2%) |
| Anglican | 119 (38·9%) | 115 (40·6%) | 234 (39·7%) |

| | Control | Intervention | All Students |
|---|---|---|---|
| Pentecostal | 45 (14·7%) | 41 (14·5%) | 86 (14·6%) |
| Seventh Day Adventist | 24 (7·8%) | 15 (5·3%) | 39 (6·6%) |
| Muslim | 34 (11·1%) | 29 (10·2%) | 63 (10·7%) |
| **Marital status** | | | |
| Single | 62 (20·1%) | 54 (19·1%) | 116 (19·7%) |
| In a relationship | 21 (6·8%) | 19 (6·7%) | 40 (6·8%) |
| Married / living together | 197 (64·0%) | 191 (67·7%) | 388 (65·8%) |
| Divorced / separated / widowed | 28 (9·1%) | 18 (6·4%) | 46 (7·8%) |
| **Housing** | | | |
| Own | 130 (42·2%) | 101 (35·7%) | 231 (39·1%) |
| Rented | 90 (29·2%) | 72 (25·4%) | 162 (27·4%) |
| Live somewhere without paying | 20 (6·5%) | 27 (9·5%) | 47 (8·0%) |
| Employer pays | 67 (21·8%) | 81 (28·6%) | 148 (25·0%) |
| Other | 1 (0·3%) | 2 (0·7%) | 3 (0·5%) |

***Notes**: All statistics are n (%) unless otherwise specified· The quantity of missing data was low for all measures (<1%) and similar in both study arms·*

## Supplementary table 2: Subgroup analyses for student self-report past week physical violence

| | Summary statistics | | Intervention Effect | |
|---|---|---|---|---|
| | **Control (N = 1,899)** | **Intervention (N = 1,921)** | **Unadjusted Model** | **Interaction effect** |
| | % (n / N) | % (n / N) | odds ratio (95% CI) p value | p value |
| **Students' sex** | | | | |
| Male | 47·9% (422 / 881) | 27·3% (242 / 885) | 0·34 (0·21 to 0·56) p<0·0001 | p=0·0431 |
| Female | 49·3% (502 / 1018) | 34·1% (353 / 1036) | 0·46 (0·29 to 0·74) p=0·0012 | |
| **School location** | | | | |
| Rural | 48·6% (496 / 1020) | 30·1% (329 / 1093) | 0·39 (0·22 to 0·70) p=0·0014 | p=0·8755 |
| Urban | 48·7% (428 / 879) | 32·1% (266 / 828) | 0·43 (0·20 to 0·90) p=0·0247 | |
| **School level prevalence of physical violence at baseline** | | | | |
| Low (<55%) | 51·1% (428 / 837) | 31·7% (352 / 1110) | 0·41 (0·22 to 0·78) p=0·0068 | p=0·9192 |
| High (>55%) | 46·7% (496 / 1062) | 30·0% (243 / 811) | 0·39 (0·21 to 0·75) p-0·0046 | |
